# Incidence and Risk of Infection Associated With Fingolimod in Patients With Multiple Sclerosis: A Systematic Review and Meta-Analysis of 8,448 Patients From 12 Randomized Controlled Trials

**DOI:** 10.3389/fimmu.2021.611711

**Published:** 2021-03-08

**Authors:** Zhao Zhao, Chun-Lai Ma, Zhi-Chun Gu, Yue Dong, Yang Lv, Ming-Kang Zhong

**Affiliations:** ^1^Department of Pharmacy, Huashan Hospital, Fudan University, Shanghai, China; ^2^Department of Pharmacy, Renji Hospital, School of Medicine, Shanghai Jiaotong University, Shanghai, China; ^3^Department of Pharmacy, The First Affiliated Hospital, College of Clinical Medicine of Henan University of Science and Technology, Luoyang, China

**Keywords:** fingolimod, multiple sclerosis, infection, dose-dependence, meta-analysis

## Abstract

**Background and Aims:** There is a controversy regarding whether fingolimod is associated with an increased risk of infection in patients with multiple sclerosis (MS). We performed a systematic review and meta-analysis of data from randomized controlled trials (RCTs) to determine the risk of infection in these patients.

**Methods:** We systematically searched PubMed, EMBASE, the Cochrane Library, and clinicaltrials.gov from inception to April 8, 2020, to identify RCTs that reported the occurrence of infection in patients with MS treated with fingolimod. Relative risks (RRs) and 95% confidence intervals (95% CIs) were calculated using the random-effects model.

**Results:** Twelve RCTs including 8,448 patients were eligible. Compared with the control (placebo and other active treatments), fingolimod significantly increased the risk of infection (RR, 1.16; 95% CI, 1.07–1.27; *I*^2^, 81%), regardless of whether the infection was a general infection (RR, 1.14; 95% CI, 1.05–1.25; *I*^2^, 78%), or a serious infection (RR, 1.49; 95% CI, 1.06–2.10; *I*^2^, 0%). Analyses of subgroups found that fingolimod significantly increased the risk of lower respiratory infection (RR, 1.48; 95% CI, 1.19–1.85; *I*^2^, 0%) and herpes virus infection (RR, 1.34; 95% CI, 1.01–1.78; *I*^2^, 9%). There appears to be no dose-dependent increase in the risk of infection associated with fingolimod (0.5 mg: RR, 1.15; 95% CI, 1.07–1.25; *I*^2^, 91%; 1.25 mg: RR, 1.11; 95% CI, 0.97–1.28; *I*^2^, 81%; P_interaction_ = 0.66).

**Conclusions:** Compared with a placebo and other active treatments, fingolimod was associated with a 16% increase in the risk of infection, especially lower respiratory infection and herpes virus infection. The risk of infection associated with fingolimod might not be dose related.

## Introduction

Multiple sclerosis (MS) is an immune-mediated chronic inflammatory demyelinating disease that mainly affects the central nervous system. Clinically, it is characterized by recurrent relapses, progression, or both, typically striking adults, primarily young adults, and ultimately leading to severe neurological disability (1, 2).

Disease-modifying therapy (DMT), which effectively reduces the recurrence rate and the accumulation of disability, is the preferred treatment in the remission period of MS. At present, there are 15 DMTs approved by the US Food and Drug Administration (FDA), including first-generation DMTs [such as interferon beta (IFN-β) and glatiramer acetate (GA)] and second-generation DMTs (such as fingolimod, teriflunomide, alemtuzumab, ocrelizumab, daclizumab, mitoxantrone, and natalizumab) (3). All DMTs target the immune system and interfere with the inflammatory process of the disease through immunomodulation or immunosuppression, which theoretically leads to a potential risk of infection in patients with MS (4). Therefore, the infection risk due to DMT has become one of the main considerations in the clinical decision-making process.

Among the second-generation DMTs, fingolimod is the first oral DMT approved by the FDA. With the dual functions of the regulation of immune inflammation and the protection of the central nervous system, fingolimod is one of the first-line DMTs for MS (5, 6). However, safety issues associated with fingolimod in randomized controlled trials (RCTs) have raised concerns about the risk of infection. More than 80% of the subjects who were included in three large phase III clinical trials of fingolimod experienced an infection of the event during the trial. FREEDOMS I (7) and II (8) showed that there was no significant difference in the incidence of infection between the fingolimod treatment group and the control group. In the TRANSFORMS study (9), the infection rate in the fingolimod treatment group was significantly higher than that in the control group. Given the contradictory results above, we therefore summarized all available evidence from RCTs for a comprehensive and rigorous meta-analysis of the risk of infection associated with fingolimod.

## Methods

### Data Sources and Searches

We followed the standards of the Cochrane Collaboration and the Preferred Reporting Items for Systematic Reviews and Meta-Analyses (PRISMA) statement for reporting systematic reviews (10). We searched PubMed, EMBASE, and the Cochrane Library databases (up to April 8, 2020) to identify published RCTs that focused on patients with MS treated with fingolimod. The search terms were as follows: (“Multiple sclerosis” OR “Sclerosis, Multiple” OR “Sclerosis, Disseminated” OR “Disseminated Sclerosis” OR “Multiple Sclerosis” OR “Multiple Sclerosis, Acute Fulminating” OR “related-limiting Multiple Sclerosis” OR “primary progressive Multiple Sclerosis” OR “secondary progressive Multiple Sclerosis”) AND (“fingolimod” OR “FTY 720” OR “Gilenya” OR “Gilenia” OR Fingolimod) AND (“clinical trial” OR “controlled clinical trial” OR “randomized controlled trials”). We also identified potential studies from the clinicaltrials.gov platform (www.clinicaltrials.gov).

### Study Selection and Outcomes

Studies were eligible if they met the following criteria: (1) RCTs reported in full-text publications; (2) comparison of fingolimod with a placebo or other DMTs (IFN-β, GA, teriflunomide, dimethyl pimarate-DMF, natalizumab, alemtuzumab, ocrelizumab, daclizumab, mitoxantrone, etc.) in patients with MS; and (3) the infection was reported as an adverse event. Two reviewers (ZZ and YL) independently screened all citations from the initial search. Any discrepancy was referred to a third reviewer (ZG) and resolved by discussion. The primary outcome of this study was the overall infection, and the secondary outcomes were general infection, serious infection, and other different types of infection. According to the definition of serious adverse events in clinical studies on the clinicaltrials.gov website, serious infection in studies included in this meta-analysis was defined as an adverse event with the following results: (1) life-threatening or resulting in death or (2) patient hospitalization or extension of a current hospital stay, resulting in an ongoing or significant incapacity or interfering substantially with normal life functions. An infection event that did not meet the definition above was considered a general infection.

### Data Extraction

Two reviewers (ZZ and YL) independently extracted the data using a self-designed form, which included the first author (publication year), the National Clinical Trial (NCT) number, the sample size, duration of follow-up, the study design (intervention groups and control groups), the country of origin, patient characteristics (age and sex ratio), disease characteristics [MS subtype and expanded disability status scale (EDSS) criteria], DMTs used in 30 days prior to the start of the study, concomitant drugs, and data of infection events. Published data and supplementary data on the clinicaltrials.gov platform were collected for each of the studies, which included upper respiratory tract infection (nasopharyngitis, sinusitis, rhinitis, pharyngitis, etc.), lower respiratory tract or lung infection (bronchitis, pneumonia, etc.), influenza virus infection, herpes viral infection (herpes zoster infection, oral herpes infection, cerebral herpes infection, etc.), digestive system infection (appendicitis, gastroenteritis, diverticulitis, etc.), urinary system infection (urinary tract infection, cystitis, pyelonephritis, etc.), abscess (streptococcal abscess, knee abscess, abdominal abscess, etc.), and other infections, such as otitis media, urinary sepsis, cryptococcal infection, and vulvitis.

### Quality Evaluation

The methodological quality of each included RCT was assessed according to the Cochrane Collaboration Risk of Bias Tool (11). The quality of trials was judged as low, unclear, or high in terms of the risk of bias based on the following domains: random sequence generation (selection bias), allocation concealment (selection bias), blinding (performance bias and detection bias), incomplete outcome data (attrition bias), and selective reporting (reporting bias).

### Statistical Analysis

Statistical analyses were performed using RevMan 5.3 software (Nordic Cochrane Centre, The Cochrane Collaboration). Relative risks (RRs) and their 95% confidence intervals (95% CIs) were used to calculate the comparative effect sizes, with *p* < 0.05 indicating a statistically significant difference. Heterogeneity between studies was assessed and judged as low (< 25%), moderate (25–75%), and high (> 75%) by the *I*^2^ statistic (12). Subgroup analyses were performed according to the severity of infection (general infection and serious infection), different types of infection events (upper respiratory tract infection, lower respiratory tract and lung infection, influenza, herpes virus infection, digestive system infection, urinary system infection, and abscess), and the dosage of fingolimod (0.5 mg daily and 1.25 mg daily). An interaction analysis (*p* for interaction) was performed to evaluate the estimated difference between a high dosage and low dosage of fingolimod. A leave-1-out sensitivity analysis was applied to explore whether a single study had an excessive influence on infection incidence. To detect the robustness of the results, further serial sensitivity analyses were conducted by excluding studies that were an open-label design, or excluding studies whose follow-up durations were < 12 months, or excluding studies that used an active agent as the control (IFN-β, GA, natalizumab) (13). Potential publication bias was evaluated by visually inspecting the funnel plots (12).

## Results

### Search Results and Study Evaluation

**Figure 1:**
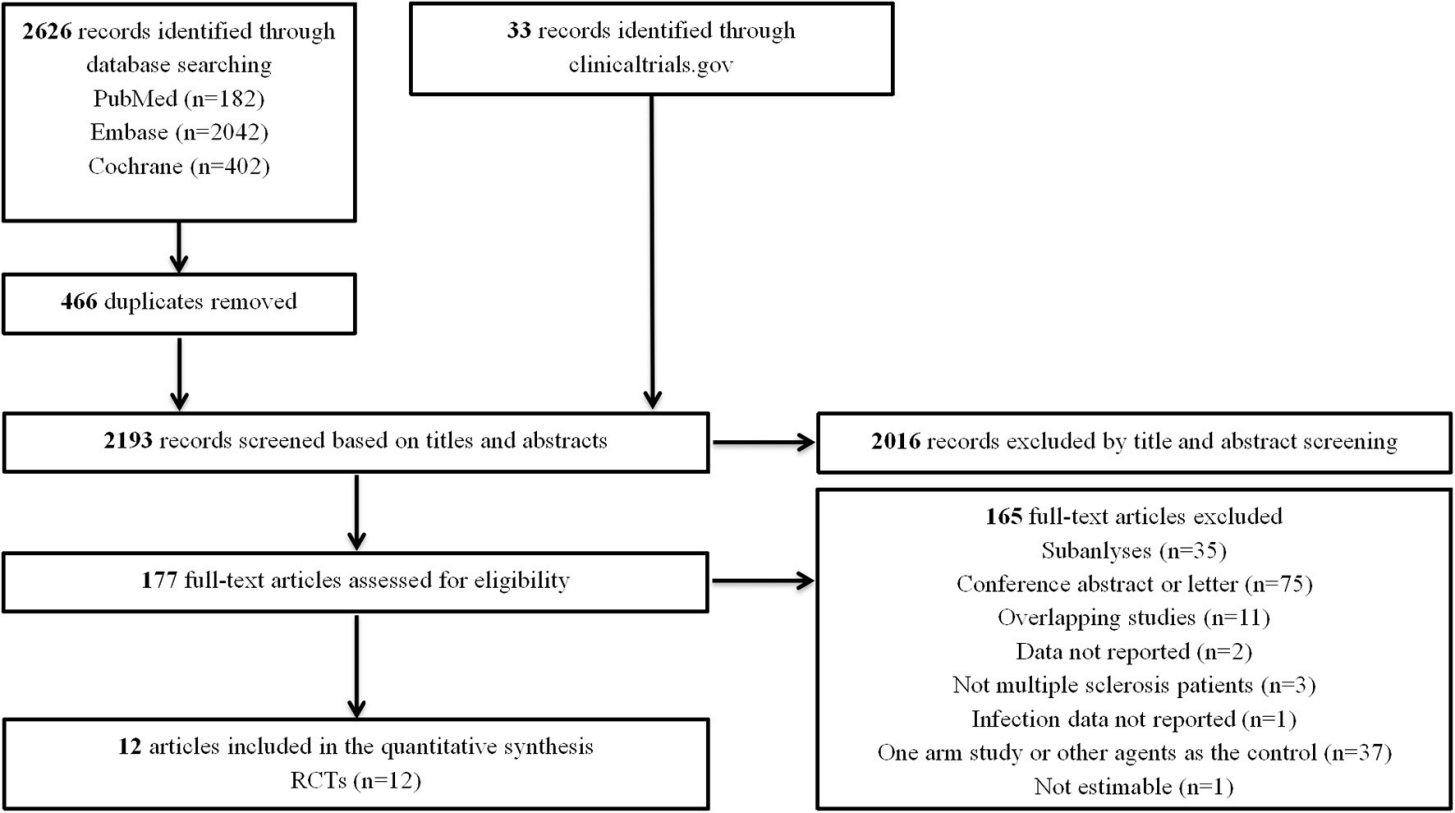
Flow diagram for the selection of eligible randomized controlled trials. RCTs, randomized controlled trials.

Our initial search identified 2,626 records from databases and 33 records from the clinicaltrials.gov platform; 2,016 records were excluded by screening titles and abstracts. Then, we reviewed the full text of the remaining 177 articles and ultimately included 12 RCTs (7–9, 14–20) (Figure 1). The characteristics of the included RCTs and the detailed infection outcomes reported in each RCT are summarized in Table 1, Supplementary Table 1. The included studies were published from 2010 to 2019, with trial durations ranging from 6 weeks to 24 months. A total of 8,448 patients were enroled, among which 5,257 (62.2%) patients were treated with fingolimod and 3,191 (37.8%) patients were treated with a placebo or first-generation DMTs. Of these 12 trials, all studies (6,508 patients) involved low-dose fingolimod (0.5 mg daily), and four studies (1,940 patients) also involved high-dose fingolimod (1.25 mg daily). Details of the quality evaluation are summarized in Supplementary Figure 1. Of the 12 RCTs, 4 were non-double-blind clinical studies (16, 20); therefore, we considered the quality of the evidence to be moderate.

**Table 1:** Characteristics of randomized clinical trials.

**Source**	**Total** **number**	**Duration**	**Trial group**		**Control group**		**Participants**		**MS subtype**	**EDSS** **criteria**	**Previous** **DMT use**	**Combination** **medicine**
			**Treatment**	***n***	**Treatment**	***n***	**Age**	**Female,%**				
Cohen et al. (9) (TRANSFORMS)	1,292	12 months	Fingolimod 0.5 mg or 1.25 mg po qd	748	IFNβ-1a 30 μg im qw	435	18–55	66.6 (fingolimod) 67.8 (placebo)	RRMS	0–5.5	NA	NA
Kappos et al. (7) (FREEDOMS)	1,272	24 months	Fingolimod 0.5 mg or 1.25 mg po qd	854	Placebo po qd	418	18–55	69.2 (fingolimod) 71.3 (placebo)	RRMS	0–5.5	NA	NA
Saida et al. (14)	171	6 months	Fingolimod 0.5 mg or 1.25 mg po qd	114	Placebo po qd	57	18–60	69.2 (fingolimod) 68.4 (placebo)	RRMS	0–6.0	NA	NA
Calabresi et al. (8) (FREEDOMS II)	1,083	24 months	Fingolimod 0.5 mg or 1.25 mg po qd	748	Placebo po qd	335	18–55	76.5 (fingolimod) 81 (placebo)	RRMS	0–5.5	NA	NA
Fox et al. (16) (EPOC)	1,053	6–9 months	Fingolimod 0.5 mg po qd	790	IFNβ-1b 0.25 mg sc qod or IFNβ-1a 30 μg im qw or IFNβ-1a 22/44 μg sc tiw or GA 20 mg sc qd	263	18–65	76.1 (fingolimod) 79.1 (iDMT)	RRMS	0–5.5	GA or IFNβ-1a or IFNβ-1b or natalizumab	NA
Kappos et al. (17)	138	12 weeks	Fingolimod 0.5 mg po qd	95	Placebo po qd	43	18–55	68.4 (fingolimod) 67.4 (placebo)	RRMS	0–6.5	NA	Seasonal influenza vaccination and tetanus booster vaccination
Lublin et al. (18) (INFORMS)	970	36 months to 2 years	Fingolimod 0.5 mg po qd	483	Placebo po qd	487	25–65	49 (fingolimod) 48 (placebo)	PPMS	0–5.0	NA	NA
Comi et al. (21) (GOLDEN)	198	18 months	Fingolimod 0.5 mg po qd	106	IFNβ-1b 0.25 mg sc every other day	51	18–60	71.25 (0.5 mg) 67.86 (IFN β-1b)	RRMS	0–5.0	NA	NA
Chitnis et al. (19) (PARADIGMS)	215	24 months	Fingolimod 0.5 mg po qd (0.25 mg po qd for patients with a body weight ≤40 kg)	107	IFNβ-1a 30 μg im qw	108	10–17	65.4 (fingolimod) 65.4 (IFN β-1a)	RRMS	0–5.0	NA	NA
Cree BAC et al. (20) (PREFERMS)	881	12 months	Fingolimod 0.5 mg po qd	436	GA 20 mg sc qd or IFNβ-1a 30 μg im qw	439	18–65	71.3 (fingolimod) 74.9 (iDMT)	RRMS	0–6.0	NA	NA
Biogen Study Medical Director (22) (REVEAL)	111	24 months	Fingolimod 0.5 mg po qd	54	Natalizumab 300 mg iv qw	54	18–65	70.4 (fingolimod) 68.5 (natalizumab)	RRMS	0–5.5	NA	NA
Novartis Pharmaceutical (23)	1,064	12 months	Fingolimod 0.25 mg or 0.5 mg po qd	722	GA 20 mg sc qd	342	18–65	74.8 (fingolimod) 73.7 (GA)	RRMS	0–6.0	NA	NA

*GA, glatirameracetate; IFNβ, interferon β; iDMT, injected disease-modifying therapy; DMT, injected disease-modifying therapy; MS, multiple sclerosis; RRMS, relapsing-remitting multiple sclerosis; PPMS, primary progressive multiple sclerosis; EDSS, expanded disability status scale; po, peros; im, intramuscular injection; sc, subcutaneous; iv, intravenous injection; qd, quaque die; qw, quaque week; qod, quaque omni die; tiw, three times per week; NA, not available*.

### Overall Risk of Infection

**Figure 2:**
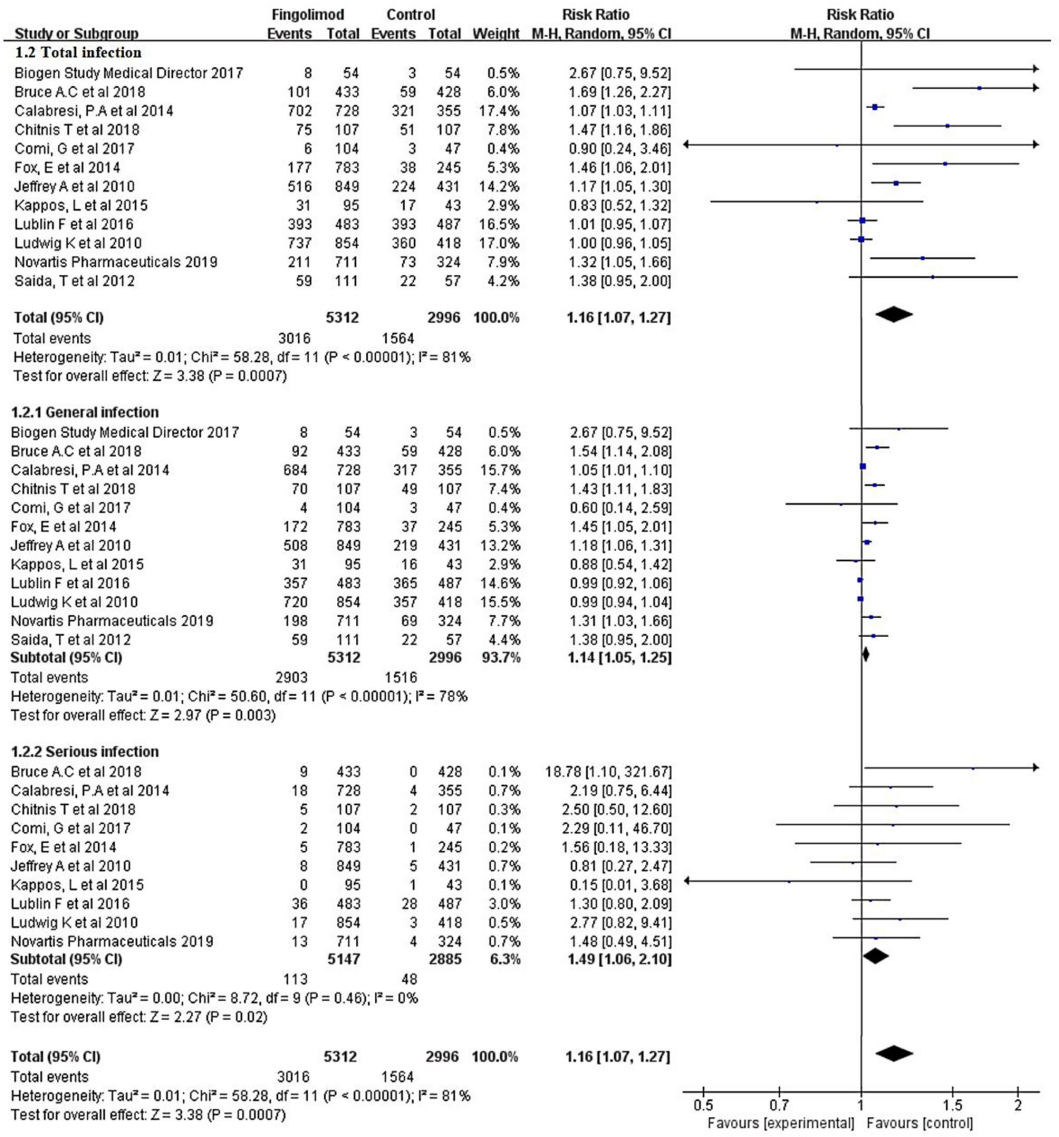
Forest plot with meta-analysis of the overall risk of infection. RR, relative risk; 95% CI, 95% confidence interval.

The overall rate of infection was 55.13% (4,580/8,308) after pooling the data from the 12 RCTs: 56.78% (3,016/5,312) in the fingolimod-treated group and 52.20% (1,564/2,996) in the control group. Compared with the control, fingolimod significantly increased the overall risk of infection (RR, 1.16; 95% CI, 1.07–1.27; *I*^2^, 81%). The subgroup analysis indicated that both general infection (RR, 1.14; 95% CI, 1.05–1.25; *I*^2^, 78%) and serious infection (RR, 1.49; 95% CI, 1.06–2.10; *I*^2^, 0%) were significantly more prevalent in the fingolimod treatment group than in the control group (Figure 2).

### Risk of Infection by Type of Infection

**Figure 3:**
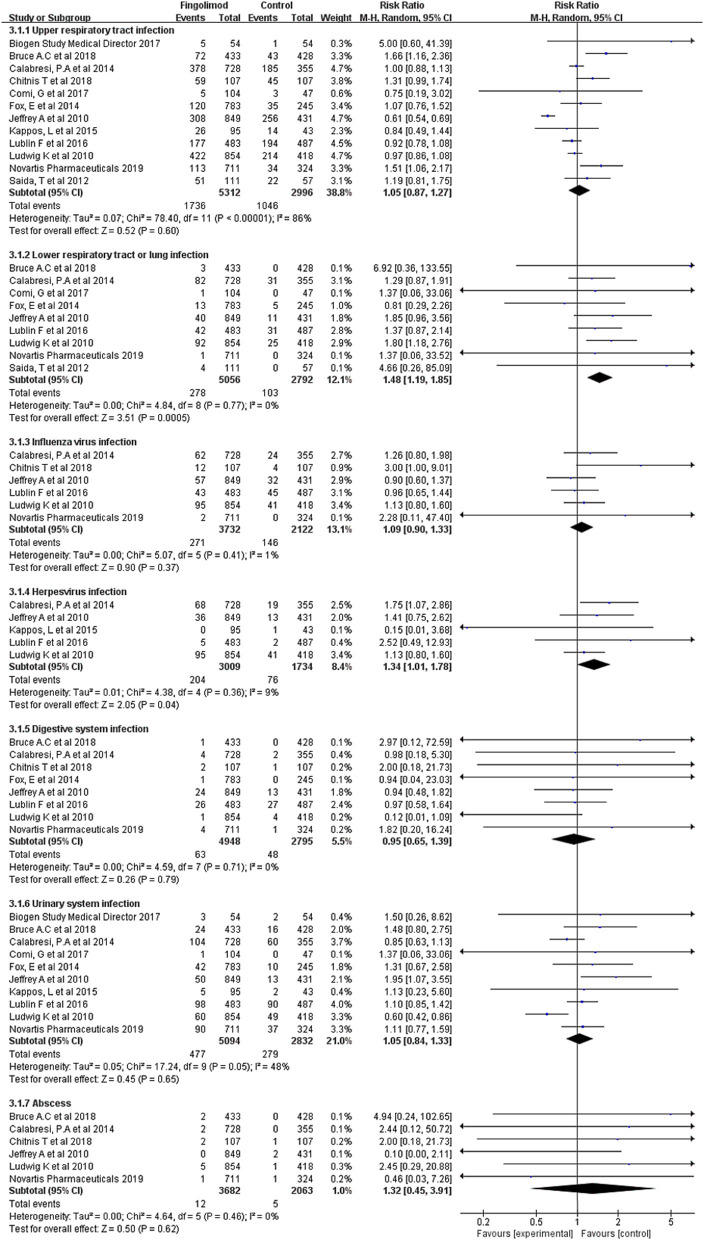
Forest plot with subgroup analysis of different types of infection. RR, relative risk; 95% CI, 95% confidence interval.

As shown in Figure 3, subgroup analyses were conducted for different infection types. Compared with the control, fingolimod significantly increased the risk of lower respiratory infection (RR, 1.48; 95% CI, 1.19–1.85; *I*^2^, 0%) and herpes virus infection (RR, 1.34; 95% CI, 1.01–1.78; *I*^2^, 9%). No significant risk difference was found between fingolimod and the control in terms of upper respiratory tract infection (RR, 1.05; 95% CI, 0.87–1.27; *I*^2^, 86%), influenza virus infection (RR, 1.09; 95% CI, 0.90–1.33; *I*^2^, 1%), digestive system infection (RR, 0.95; 95% CI, 0.65–1.39; *I*^2^, 0%), urinary system infection (RR, 1.05; 95% CI, 0.84–1.33; *I*^2^, 48%), and abscess (RR, 1.32; 95% CI, 0.45–3.91; *I*^2^, 0%).

### Risk of Infection by Dose Size

**Figure 4:**
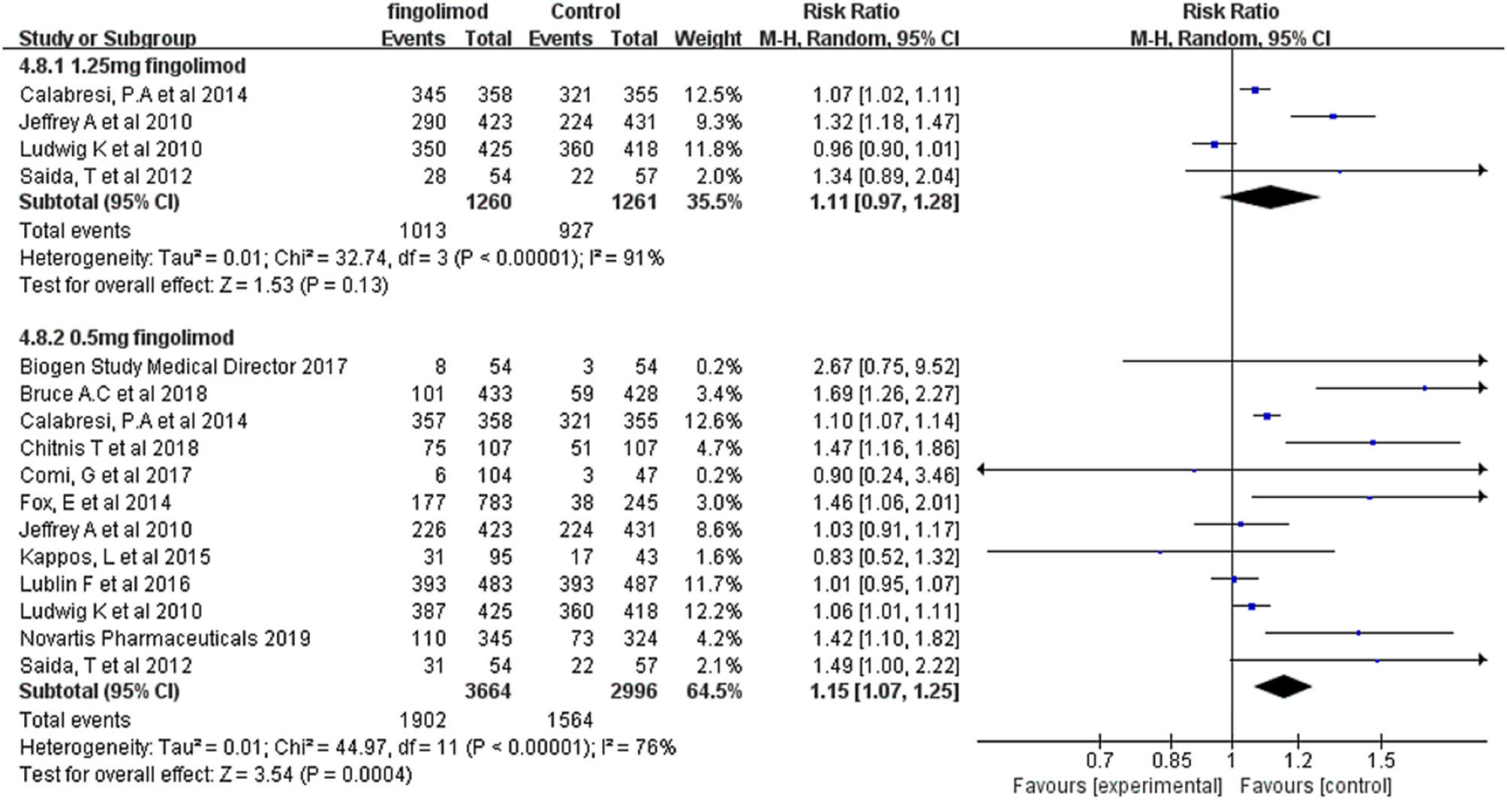
Forest plot with subgroup analysis of different doses of fingolimod for infection risk. RR, relative risk; 95% CI, 95% confidence interval.

Fingolimod was available in two doses: 0.5 mg daily and 1.25 mg daily. A total of 6,660 patients from 12 RCTs received fingolimod 0.5 mg daily, and compared with those in the control group, these patients had a significantly higher risk of infection (RR, 1.15; 95% CI, 1.07–1.25; *I*^2^, 81%). A total of 2,521 patients from four RCTs received fingolimod 1.25 mg daily, and the incidence of infection was 80.40% (1,013/1,260) in the fingolimod-treated group and 73.51% (927/1,261) in the control group, indicating that there was no significant difference in the occurrence rate of infection between the fingolimod and control groups (RR, 1.11; 95% CI, 0.97–1.28; *I*^2^, 91%) (Figure 4). However, we failed to find an estimated difference between the high dosage and low dosage of the fingolimod groups (*P*_*interaction*_ = 0.66), which indicated that the risk of infection associated with fingolimod might not be dose dependent.

### Sensitivity Analyses

The leave-1-out sensitivity analysis failed to identify any individual trial as having influenced the primacy outcome. Besides, further sensitivity analyses by excluding studies that were an open-label design or whose follow-up durations were < 12 months or that used an active agent as the control (IFN-β, GA, natalizumab), which all confirmed the robustness of primacy results (Supplementary Table 2).

### Publication Bias

Visual inspection of the funnel plots for the analyses showed that all plots exhibited fairly symmetrical inverted funnel shapes, suggesting that publication bias was not a concern (Supplementary Figure 2).

## Discussion

### Major Findings

The risk of infection has been recognized as one of the main considerations when choosing appropriate DMT for patients with MS in the clinical setting (24). As a highly effective second-generation DMT, fingolimod has great clinical application in patients with MS, but whether fingolimod increases the risk of infection remains uncertain. This systematic review and meta-analysis firstly provided a comprehensive overview of fingolimod-associated infection risk based on 12 RCTs, including 8,448 patients with MS. The major findings were as follows: (1) fingolimod use increased the risk of overall infection by 16%, and the incidence of both general and serious infections increased significantly; (2) fingolimod use was associated with a higher risk of lower respiratory and herpes virus infection; and (3) the risk of infection associated with fingolimod might be dose independent.

### Comparison With Previous Studies

There were three systematic reviews that focused on fingolimod, two of which systematically reviewed real-world data on fingolimod to determine its persistence and efficacy (25, 26). In these studies, the overall incidence of adverse events (AEs) was counted, and no systematic analysis for specific adverse events (such as infection) was performed. At present, only one study in 2019 evaluated the efficacy and safety of fingolimod using 10 RCTs (27). In that study, bronchitis, nasopharyngitis, sinusitis, and urinary tract infection were considered infection events, and the analysis indicated that fingolimod was associated with a significantly increased risk of bronchitis, which was consistent with our result that fingolimod increased the risk of lower respiratory infection. However, they did not find any significant difference between the fingolimod and control groups in terms of the overall incidence of infection. Considering an important limitation of that study, i.e., that only some AEs with a high incidence were retrieved, and their assessment of the risk of infection was not comprehensive. Therefore, considering the limitations of previous studies, this systematic review included all the infection events reported in RCTs, regardless of whether they were common or not, to systematically evaluate the risk of infection associated with fingolimod. Moreover, in addition to the currently published studies, we also included unpublished RCTs on the clinicaltrials.gov website to make the study more comprehensive.

We ultimately retrieved data from 12 RCTs, and we confirmed that fingolimod is associated with a relatively higher risk of infection than placebos and other active DMTs (IFN-β, GA, and natalizumab), which is consistent with two previous observational studies (28, 29). This study also highlighted the existence of different risk profiles for different types of infection associated with fingolimod. Subgroup analysis indicated that the incidence of lower respiratory infection and herpes virus infection increased significantly in patients treated with fingolimod. Since there were some studies suggesting that the occurrence of AEs associated with fingolimod might be dose dependent (30), we also assessed the correlation between infection risk and fingolimod dosage. However, the results showed that the risk of infection associated with fingolimod might be dose independent (*P*_interaction_ = 0.66).

### Potential Mechanism

There were two possible explanations for why fingolimod was associated with a higher risk of infection in MS patients. First, as a sphingosine-1-phosphate (S1P) analog and a functional modulator of the S1P receptor, fingolimod-P causes the internalization of S1P_1_R from the cell membrane in lymph node T cells. As a result, the functional balance between S1P_1_R and lymph node-homing CC chemokine receptor (CCR7) is interrupted, and CCR7 + primitive T cells and central memory T cells (TCM) are unable to resist CCR7-mediated lymph node retention, thereby remaining in lymph nodes. Therefore, the number of peripheral T cells migrating to the CNS decreases, which may cause the occurrence of infection (5, 31, 32). A subanalysis of a phase III RCT for fingolimod indicated that the lymphocyte count dropped rapidly within 2 weeks after the start of treatment; however, no trend was found in the relationship between the incidence of infection and decreases in lymphocytes and the duration of treatment (33). Additionally, it is worth noting that the counts of peripheral lymphocytes reflect only 2% of the total lymphocytes in the body, and fingolimod mainly reduces circulating CD4 + T cells, retaining CCR7—effector T cells involved in controlling microbial infections (34). Accordingly, the relationship between the decrease in peripheral lymphocyte counts and the infection caused by fingolimod is still controversial. Second, studies have also argued that fingolimod induces some important functional changes in the immune system, which leads to an increased risk of infection. Under the effect of fingolimod, T cells decrease the production of cytokines, such as IFN-γ, IL-17, GM-CSF, and TNF-α, which can help to effectively kill congenital effector cells (such as neutrophils and macrophages) and promote the differentiation of T cells. Additionally, in the long term, the ratios of CD4 and CD8 in patients with MS taking fingolimod show a striking reversal of the normal 2:1 ratio, reminiscent of the changes associated with AIDS. Of course, the effect of fingolimod on the immune system is by no means comparable to that of AIDS-associated immune changes, but the effect of the reversal caused by fingolimod on the immune response is not fully understood (35). In short, the specific mechanism of fingolimod-associated infections is not yet clear, and further research and analyses are still needed.

### Clinical Considerations

Given the higher incidence of infection in patients with MS treated with fingolimod, it might be reasonable to triage patients according to the following steps: First, clinicians should conduct a comprehensive assessment of patient conditions for the possible risk factors, such as their history of infection, history of immunosuppressive exposure, vaccination history, age, etc., to determine the best DMT for individual patients (30). Second, a higher risk of herpes virus infection associated with fingolimod was indicated in this study; thus, herpes virus serology should be performed before the start of fingolimod treatment, and flu vaccination can also be considered. Third, during the treatment with fingolimod, clinicians need to be alert to the occurrence of any infection with strict monitoring of clinical signs/symptoms, especially the lower respiratory infection and herpes virus infection (28). Adequate laboratory and instrumental tests are also necessary to make an early diagnosis and promptly start the treatment where appropriate. Although the current evidence indicates that the increased risk of infection caused by fingolimod is associated with its effect on the immune function to a certain degree, there is no well-established monitoring method in clinical practice. Monitoring of peripheral lymphocyte counts can be considered for the decreases in the lymphocyte count associated with fingolimod. Suppose the lymphocyte count drops below 0.2 × 10^9^/L at any visit (at 2 weeks, 1, 2, and 3 months, and every 3 months after that). In that case, fingolimod should be temporarily discontinued for immune reconstruction (36), but this indicator has not been used as a standard for discontinuation in clinical practice. Moreover, for the potential immune downregulation of fingolimod, live attenuated vaccines should be avoided during treatment, if possible (34). Summarily, understanding the infectious effects of fingolimod, taking into account the prevention, is preferable to treatment.

### Strengths and Limitations

The major strength of this study was that we comprehensively assessed the risk of infection of patients with MS treated with fingolimod on the basis of evidence from RCTs. Certainly, there are inherent limitations in this meta-analysis. First, four RCTs included were open-label studies with low quality, although the sensitivity analysis showed that their effect on the final result was not significant. Second, the heterogeneity among the included RCTs was relatively high. To address this issue, the random-effects model was used for the meta-analyses. Besides, several subgroup analyses as well as serial sensitivity analyses were performed to strengthen the robustness of the results. All results were in line with the primacy results. Third, since the FDA only approved the clinical use of the 0.5 mg daily dose, clinical trials of 1.25 mg daily doses were limited. Hence, the analysis of different doses in our study might be limited by the small number of cases in the high-dose group; therefore, the results must be interpreted cautiously. Fourth, the clinical trials included in our study were performed at various international institutions, which might have varying expertise and ability to detect infection, making it possible that the reported incidence was biased. Fifth, the timing of infection occurrence might be related to the duration of treatment. We also conducted a sensitivity analysis by excluding short follow-up studies, and the result was consistent with primacy analyses. Sixth, due to the limited number of cases, certain infections (encephalitis viral, clostridial infection, mastoiditis, otitis media acute, urosepsis, tinea pedis, vulvitis, Lyme disease, labyrinthitis, hepatitis C, myelitis, septic shock, systemic mycosis, arthritis bacterial, clostridium difficile colitis, device-related sepsis, meningitis fungal, sepsis, etc.) were not included in the subgroup of different types of infection in the meta-analysis. Finally, this study only evaluated the infection risk of fingolimod based on the data from RCTs; to extend RCT findings to large patient populations in real-world clinical practice, further design of real-world studies on the evaluation of fingolimod safety is necessary.

## Conclusion

In conclusion, by systematically evaluating evidence from RCTs, we confirmed that fingolimod significantly increased the risk of infection, especially lower respiratory infection and herpes virus infections, in patients with MS. Both general infection and serious infection increased to varying degrees. However, the risk of infection associated with fingolimod might not be dose related. These findings can help clinicians assess the risk of infection of patients treated with fingolimod.

## Data Availability Statement

The original contributions presented in the study are included in the article/Supplementary Materials, further inquiries can be directed to the corresponding author/s.

## Author Contributions

ZZ contributed to the review of published papers, the conception of the study, the data acquisition, analysis, and interpretation, and the writing of the manuscript. C-LM and Z-CG are the guarantors of the entire study, contributed to the conception of the study, the interpretation of the data, and review of the manuscript. YD contributed to the conception of the study and review of the manuscript. YL contributed to data acquisition for the study. M-KZ supervised the study. All authors contributed to the article and approved the submitted version.

## Conflict of Interest

The authors declare that the research was conducted in the absence of any commercial or financial relationships that could be construed as a potential conflict of interest.
