# Supplementary material for: Incidence and Risk of Infection Associated With Fingolimod in Patients With Multiple Sclerosis: A Systematic Review and Meta-Analysis of 8,448 Patients From 12 Randomized Controlled Trials
Source: Front Immunol. 2021 Mar 8;12:611711. doi: 10.3389/fimmu.2021.611711 (PMC7982402; doi:10.3389/fimmu.2021.611711)
Supplement: Supplementary file 1 [file Data_Sheet_1.DOCX]

Supplementary Material

**Table S1. Detailed infection events reported in each trial**

| **Source** | **Reported infection events** |
| --- | --- |
|  |  |
| Jeffrey, A et al, 2010 (TRANSFORMS) | Upper respiratory tract infection (Nasopharyngitis, Upper respiratory tract infection, Sinusitis, Pharyngitis); Lower respiratory tract or lung infection ( Bronchitis); Influenza virus infection; Herpesvirus infection (Oral Herpes); Digestive system infection (Gastroenteritis, Appendicitis); Urinary system infection (Urinary tract infection); Abscess (Bartholin's abscess, Incision site abscess); Other infection (Administration site infection, Encephalitis viral) |
| Ludwig, K et al, 2010 (FREEDOMS) | Upper respiratory tract infection (Nasopharyngitis, Sinusitis, Acute sinusitis, Pharyngitis, Rhinitis, Pharyngotonsillitis, Tonsillitis); Lower respiratory tract or lung infection ( Bronchitis, Pneumonia); Influenza virus infection; Herpesvirus infection; Digestive system infection (Gastroenteritis, Appendicitis); Urinary system infection (Urinary tract infection, Cystitis, Pyelonephritis, Pyelonephritis acute, Pyelonephritis chronic); Abscess (Abscess jaw, Anal abscess, Bartholin's abscess, Incision site abscess, Peritoneal abscess, Streptococcal abscess, Tooth abscess); Other infection (Clostridial infection, Mastoiditis, Otitis media acute, Urosepsis) |
| Saida, T et al, 2012 | Upper respiratory tract infection (Nasopharyngitis, Pharyngitis); Lower respiratory tract or lung infection (Bronchitis); Influenza virus infection; Other infection (Tinea pedis) |
| Calabresi, P.A et al, 2014 (FREEDOMS II) | Upper respiratory tract infection (Nasopharyngitis, Upper respiratory tract infection, Sinusitis, Acute sinusitis); Lower respiratory tract or lung infection (Bronchitis, Pneumonia, Lower respiratory tract infection fungal); Influenza virus infection; Herpesvirus infection; Digestive system infection (Gastroenteritis, Appendicitis, Diverticulitis); Urinary system infection (Urinary tract infection, Kidney infection, Pyelonephritis); Abscess (Cellulitis, Streptococcal abscess); Other infection (Vulvitis, Mastoiditis, Otitis media acute, Lyme disease, Labyrinthitis, Hepatitis C) |
| Fox, E et al, 2014 (EPOC) | Upper respiratory tract infection (Nasopharyngitis, Sinusitis, Nasopharyngitis); Lower respiratory tract or lung infection (Bronchitis, Pneumonia); Digestive system infection (Appendicitis); Urinary system infection (Urinary tract infection, Kidney infection); Other infection (Urosepsis) |
| Kappos, L et al, 2015 | Upper respiratory tract infection (Nasopharyngitis, Upper respiratory tract infection, Sinusitis, Acute sinusitis); Herpesvirus infection; Urinary system infection (Urinary tract infection) |
| Lublin, F et al, 2016 (INFORMS) | Upper respiratory tract infection (Nasopharyngitis, Upper respiratory tract infection, Viral upper respiratory tract infection, Tonsillitis); Lower respiratory tract or lung infection (Bronchitis, Pneumonia, Bronchopneumonia, Tracheobronchitis); Influenza virus infection; Herpesvirus infection; Digestive system infection (Gastroenteritis, Appendicitis, Enterocolitis bacterial, Diverticulitis); Urinary system infection (Urinary tract infection, Cystitis OR bacterial cystitis, Pyelonephritis, Pyelonephritis acute); Other infection (Urosepsis, Myelitis, Septic shock, Systemic mycosis) |
| Comi, G et al, 2017 (GOLDEN) | Upper respiratory tract infection (Nasopharyngitis); Lower respiratory tract or lung infection (Bronchopneumonia); Urinary system infection (Urinary tract infection, Kidney infection, Pyelonephritis) |
| Chitnis, T et al, 2018 (PARADIGMS) | Upper respiratory tract infection (Nasopharyngitis, Upper respiratory tract infection, Viral upper respiratory tract infection, Rhinitis, Viral pharyngitis); Influenza virus infection; Digestive system infection (Appendicitis, Gastritis viral, Gastrointestinal infection); Abscess (Abscess oral, Cellulitis, Paronychia) |
| Bruce, A.C et al, 2018 (PREFERMS) | Upper respiratory tract infection (Nasopharyngitis, Sinusitis); Lower respiratory tract or lung infection (Pneumonia); Digestive system infection (Campylobacter gastroenteritis); Urinary system infection (Urinary tract infection, Pyelonephritis acute); Abscess (Tooth abscess, Cellulitis); Other infection (Bacteraemia, Viral infection) |
| Biogen Study Medical Director, 2017 (REVEAL) | Upper respiratory tract infection (Upper respiratory tract infection); Urinary system infection (Urinary tract infection) |
| Novartis Pharmaceutical, 2019 | Upper respiratory tract infection (Bronchitis, Upper respiratory tract infection, Rhinovirus infection); Lower respiratory tract or lung infection (Pneumonia); Digestive system infection (Appendicitis, Clostridium difficile colitis, Gastroenteritis, Diverticulitis, Gastroenteritis viral); Influenza virus infection (Influenza, Parainfluenzae virus infection); Urinary system infection (Urinary tract infection); Abscess (Extradural abscess, Cellulitis); Other infection (Arthritis bacterial, Clostridium difficile colitis, Device related sepsis, Meningitis fungal, Sepsis) |

**Table S2. Sensitivity analyses with meta-analysis of the risk of infection**

| **Study Omitted** | **RR** | **95%CI** |
| --- | --- | --- |
| Biogen Study Medical Director, 2017 (REVEAL) | 1.16 | 1.06-1.26 |
| Bruce A.C et al, 2018 (PREFERMS) | 1.12 | 1.04-1.21 |
| Calabresi, P.A et al, 2014 (FREEDOMS II) | 1.21 | 1.07-1.37 |
| Comi, G et al, 2017 (GOLDEN) | 1.13 | 1.04-1.23 |
| Chitnis T et al, 2018 (PARADIGMS) | 1.16 | 1.07-1.27 |
| Fox, E et al, 2014 (EPOC) | 1.14 | 1.05-1.24 |
| Jeffrey A et al, 2010 (TRANSFORMS) | 1.16 | 1.06-1.27 |
| Kappos, L et al, 2015 | 1.18 | 1.07-1.29 |
| Ludwig K et al, 2010 (FREEDOMS) | 1.21 | 1.09-1.35 |
| Lublin F et al,2016 (INFORMS) | 1.22 | 1.09-1.36 |
| Novartis Pharmaceuticals, 2019 | 1.14 | 1.05-1.25 |
| Saida, T et al, 2012 | 1.15 | 1.06-1.26 |
| **Excluding studies that were open-label design** | 1.10 | 1.02-1.18 |
| **Excluding studies that follow-up durations were less than 12 months** | 1.14 | 1.05-1.25 |
| **Excluding** studies that used IFN-β as the comparator | 1.12 | 1.03-1.23 |
| **Excluding** studies that used IFN-β or GA as the comparators | 1.10 | 1.02-1.18 |
| Excluding studies that used natalizumab as the comparator | 1.16 | 1.06-1.26 |
| Excluding studies that used GA as the comparator | 1.14 | 1.05-1.25 |

RR: relative risk; CI: confidence interval; GA: glatiramer acetate; studies of **open-label design** including Biogen Study Medical Director, 2017 (REVEAL), Bruce A.C et al, 2018 (PREFERMS), Comi, G et al, 2017 (GOLDEN), and Fox, E et al, 2014 (EPOC); studies that follow-up duration were less than 12 months including Saida, T et al, 2012, Kappos, L et al, 2015, and Fox, E et al, 2014 (EPOC); studies that used IFN-β as the comparator including Comi, G et al, 2017 (GOLDEN ), Chitnis T et al, 2018 (PARADIGMS), Jeffrey A et al,2010 (TRANSFORMS); studies that used IFN-β or GA as the comparators including Fox, E et al, 2014 (EPOC) and Bruce A.C et al, 2018 (PREFERMS); studies that used Natalizumab as the comparator including Biogen Study Medical Director, 2017 (REVEAL); studies that used GA as the comparator including Novartis Pharmaceuticals, 2019.

**Figure S1.** Risk of bias summary：review of authors’ judgments about each risk of bias item for each included study. + indicates low risk; , high risk; ?, unclear risk.

**
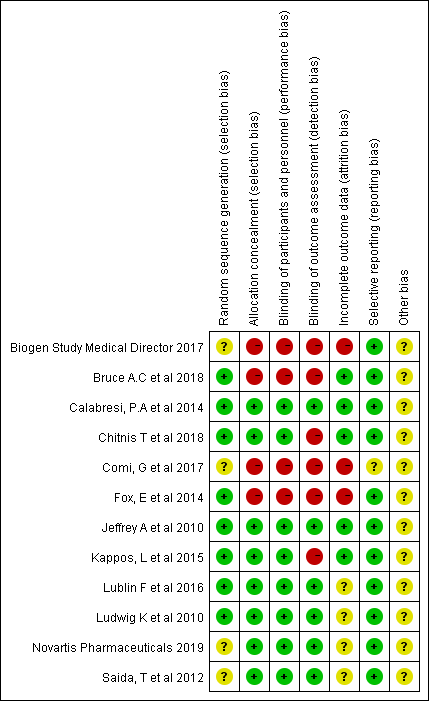
**

**Figure S2.** Funnel plot to assess publication bias

**
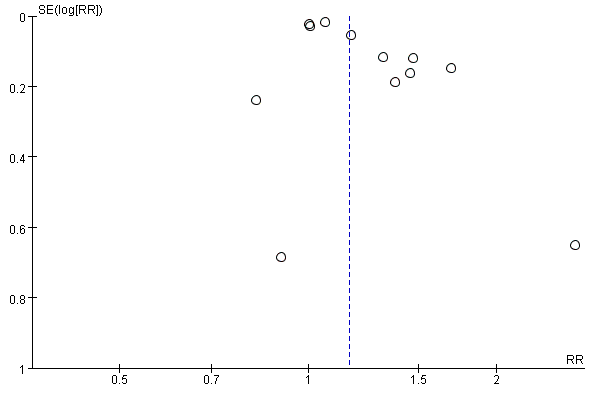
**

1. **Total RCTs**

**
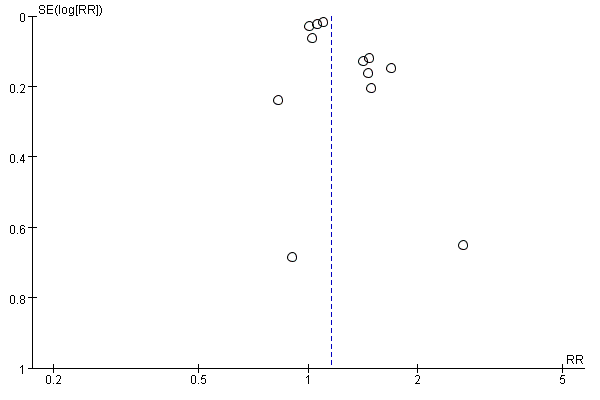
**

**(B) 0.5 mg/daily of Fingolimod**
